# Supplementary material for: AI reveals insights into link between CD33 and cognitive impairment in Alzheimer’s Disease
Source: PLoS Comput Biol. 2023 Feb 13;19(2):e1009894. doi: 10.1371/journal.pcbi.1009894 (PMC9956604; doi:10.1371/journal.pcbi.1009894)
Supplement: S1 Note — (PDF) [file pcbi.1009894.s005.pdf]

Supplementary Note S1: iVAMBNs Module Definition

The modules for the VAMBN network are defined through the clustering of the knowledge graph. There were two exceptions: First, modules 4 and 5 enrich the same NeuroMMSig mechanism, namely, TGF-beta signaling, and were thus merged into one module for modeling. The second exception is based on the unavailability of some genes in the gene expression data: Out of the total of 383 genes in the knowledge graph, only 330 were expressed in all AD cohort studies, resulting in seven modules consisting of only one gene after mapping to the gene expression data. Therefore, these genes were used as standalone genes in the VAMBN approach. Altogether there were eight standalone genes: CASP7, CD33, DLG4, GRIN1, NAV3, PPARG, REL and TRAF1. Genes HSPB2, HSPB3 and MIR101-1 were not be measured in all datasets.

On top of modules comprising molecular mechanisms, one *phenotype* module summarizes MMSE scores and Braak stages and some demographic features, namely age, gender, years of education, the APOE genotype, and the brain region are integrated as standalone features.

After module definition, the next step in iVAMBN model generation, is the training of the auto-encoded values per module. Therefore, a HI-VAE was trained for each module separately. A grid search was used to find the best hyperparameters, and each candidate hyperparameter set was evaluated via a 3-fold cross validation. Once finished with this process, each module dimension was reduced to one.
