# Supplementary material for: AI reveals insights into link between CD33 and cognitive impairment in Alzheimer’s Disease
Source: PLoS Comput Biol. 2023 Feb 13;19(2):e1009894. doi: 10.1371/journal.pcbi.1009894 (PMC9956604; doi:10.1371/journal.pcbi.1009894)
Supplement: S2 Note — (PDF) [file pcbi.1009894.s006.pdf]

Supplementary Note S2: iVAMBNs Knowledge Integration

The encoded values from the HI-VAEs are used for training of a Modular Bayesian Network. Part of this training is a structure training, which was performed following three different strategies with five scenarios in total. This gives the opportunity to investigate the level of knowledge integration within the Modular Bayesian Network construction. The three different strategies for structure learning are: 1) completely data driven, 2) knowledge informed, and 3) completely knowledge driven. The second one, knowledge informed, was realized in three different scenarios. The differences between all these options are realized with various black- and white list definitions: while there is no restriction of edges between genes in the completely data driven scenario, the whole network structure is pre-defined in the completely knowledge driven one. More details about the defined blacklists and the definition of the different scenarios can be found in the Methods Section. Additionally, to the different scenarios, various structure learning algorithms were tested that are available in the bnlearn package, including

- constraint-based algorithms: PC, Grow-Shrink (GS), Incremental Association (IAMB), Fast Incremental Association (Fast-IAMB), Interleaved Incremental Association (Inter-IAMB), Incremental Association with FDR (IAMB-FDR), Max-Min Parents and Children (MMPC), Semi-Interleaved HITON-PC and Hybrid Parents and Children (HPC),
- score-based approaches: hill-climbing (HC) and Tabu search (TABU),
- hybrid approaches: Max-Min Hill Climbing (MMHC), Hybrid HPC (H2PC), and 2-phase Restricted Maximization (RSMAX2).

Once done for every scenario, the structure learning was evaluated based on the 10-fold cross-validated loss on held out test data. It should be noted, that the testing of constrained-based algorithms for no knowledge integration and the knowledge informed approach resulted in errors and therefore could not be evaluated. The plots include only those options that could be evaluated in the 10-fold cross-validation scenario. Additionally, constrained-based and hybrid approaches do not allow to set at graph as initialization, so that only the first option of the knowledge informed strategy was integrated in the analysis.

As shown in Fig **A**, the completely knowledge driven approach, which forces the BN to have

the fixed structure of the knowledge graph, does perform much worse than all other approaches for all structure learning algorithms. Hill climbing (HC) and Tabu search are performing best for both the knowledge informed and completely knowledge driven approach.

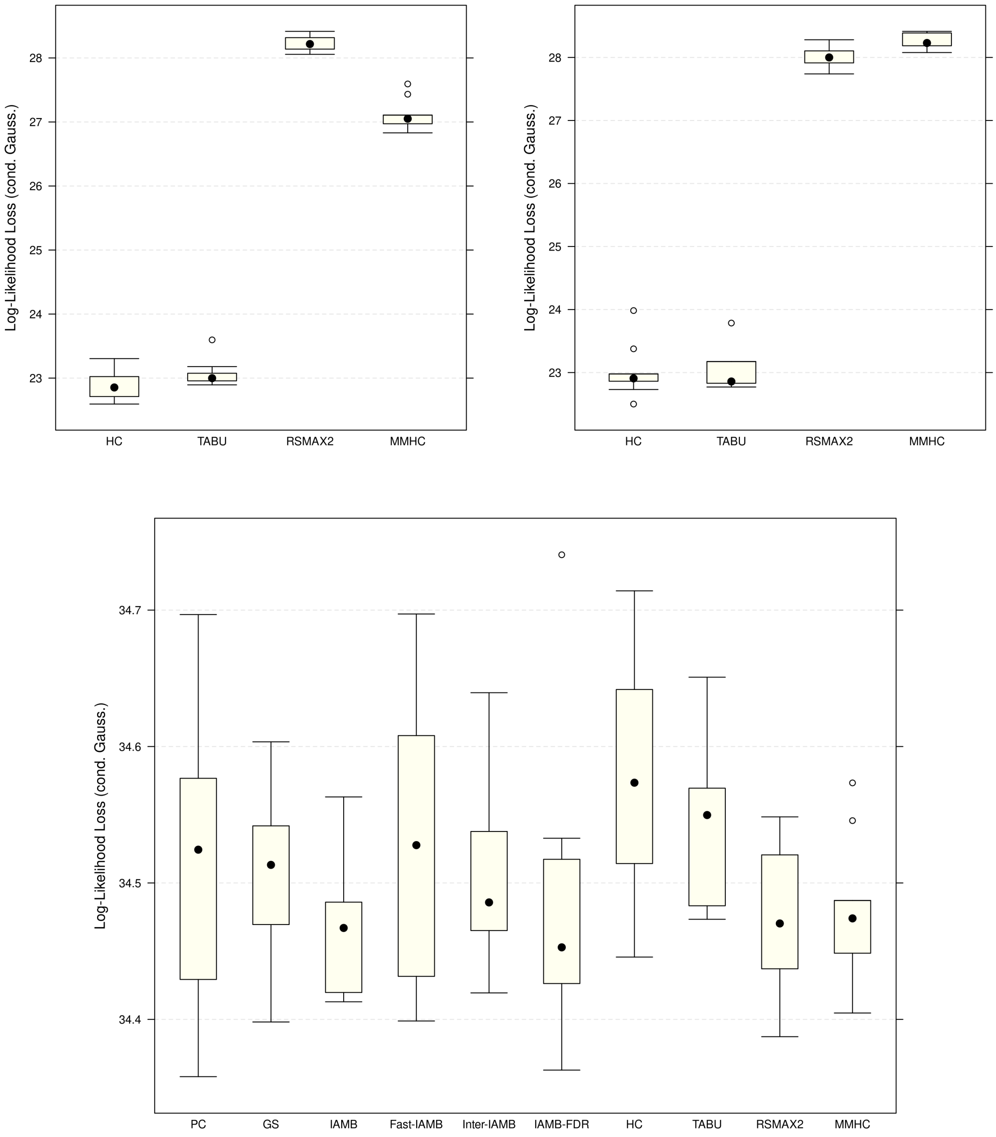

Fig A. Log likelihood loss (i.e. negative log-likelihood) of different knowledge integration strategies and structure learning algorithms. Completely knowledge driven approach (bottom), forcing the BN to have the fixed structure of the knowledge graph, does perform much worse than the completely data driven (upper left) or knowledge informed (upper right) approaches for all structure learning algorithms. Hill climbing (HC) and Tabu search are performing best for both the knowledge informed and completely knowledge driven approach.

Based on these results we evaluated the hill climbing and Tabu search algorithm for all the five scenarios as shown in **B**. The lowest average cross-validated loss could be observed for the second and third option of the knowledge informed approach for both the hill climbing and Tabu search. For further processing we arbitrary chose the hill climbing algorithm as our structure learning algorithm and the third option of the knowledge informed approach, which forces the KG edges' to be present in the BN, but allows for training of new edges, while using the KG also as the initialization for structure learning. Therefore, we went ahead with this strategy for all further analyses.

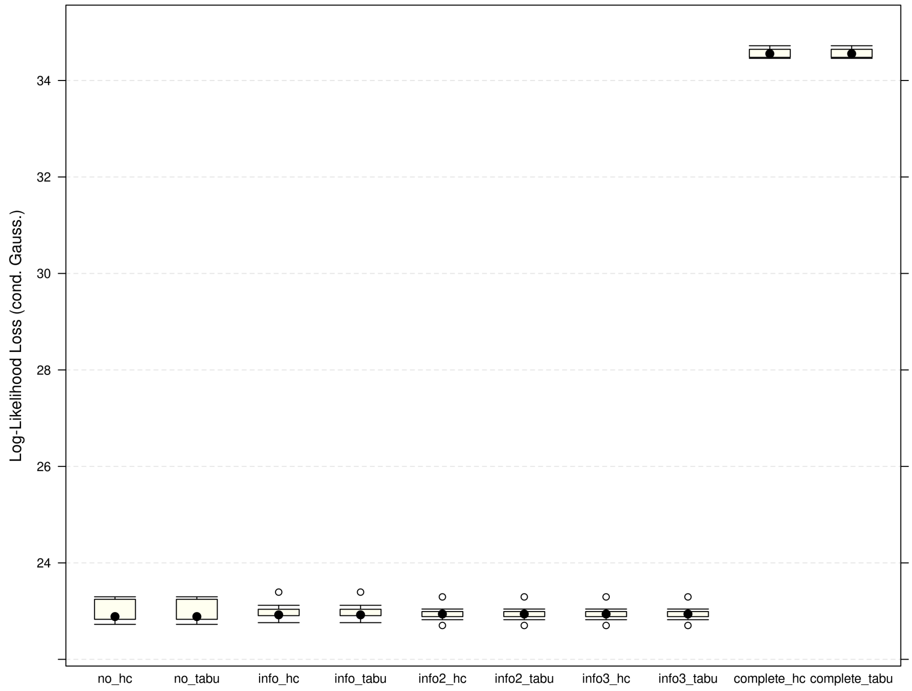

Fig B. Log likelihood loss (i.e. negative log-likelihood) of different knowledge integration strategies for structure learning with hill climbing and Tabu search. Completely knowledge driven approach, forcing the BN to have the fixed structure of the knowledge graph, does perform much worse than the completely data driven or knowledge informed approaches for both the hill climbing (HC) and Tabu search. One can observe the lowest average log likelihood loss for the second and third option of the knowledge informed approach for both the hill climbing and Tabu search.
