# Supplementary material for: AI reveals insights into link between CD33 and cognitive impairment in Alzheimer’s Disease
Source: PLoS Comput Biol. 2023 Feb 13;19(2):e1009894. doi: 10.1371/journal.pcbi.1009894 (PMC9956604; doi:10.1371/journal.pcbi.1009894)
Supplement: S3 Note — (PDF) [file pcbi.1009894.s007.pdf]

Supplementary Note S3: Specificity and Sensitivity Analysis

Specificity to brain region

To understand the specificity of our iVAMBN model to the cortical brain region we analyzed normalized gene expression data from different brain regions from ROSMAP and Mayo. Within ROSMAP these data have been measured in separate batches. To account for the batch effects we thus normalized samples against the original training data via ComBat [1]. Still, we observed a considerable grouping of ROSMAP samples into two clusters after applying ComBat (Fig **A**). Hence, we decided to analyze samples from both clusters separately. An overview of all the used datasets is provided in Table **A**.

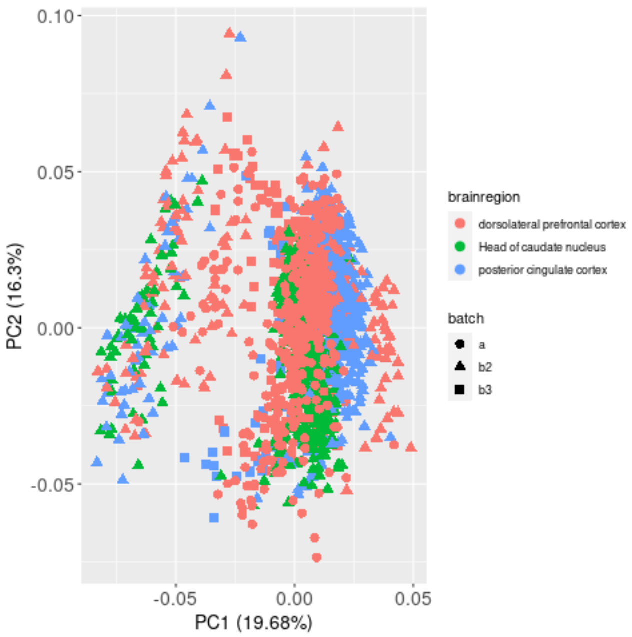

Fig A. PCA of Combat corrected data. Included were all samples from the ROSMAP data (a: primary analysis, batch 2 (b2) and batch 3 (b3)). Colored are the different brain regions the samples were taken from. For the following analysis, cluster 1 is defined with the samples on the left side of the plot and cluster 2 from those samples grouped on the right side of the plot.

Table A. Overview of samples. The table shows the number of samples per study (ROSMAP or Mayo), brain region, cluster (if applicable), and indication (healthy controls (HC) or Alzheimer's Disease (AD)), that were used in the primary analysis, for external validation, and for specificity analyses.

source	brain region	cluster	indication	number samples	used for
ROSMAP	dorsolateral prefrontal cortex		AD	221	primary
ROSMAP	dorsolateral prefrontal cortex	cluster 1	AD	158	brain region specificity
ROSMAP	dorsolateral prefrontal cortex	cluster 2	AD	249	brain region specificity
ROSMAP	dorsolateral prefrontal cortex	cluster 1	HC	97	disease specificity
ROSMAP	dorsolateral prefrontal cortex	cluster 2	HC	274	disease specificity
ROSMAP	head of caudate nucleus	cluster 1	AD	97	brain region specificity
ROSMAP	head of caudate nucleus	cluster 2	AD	153	brain region specificity
ROSMAP	head of caudate nucleus	cluster 1	HC	83	disease specificity
ROSMAP	head of caudate nucleus	cluster 2	HC	165	disease specificity
ROSMAP	posterior cingulate cortex	cluster 1	AD	70	brain region specificity
ROSMAP	posterior cingulate cortex	cluster 2	AD	151	brain region specificity
ROSMAP	posterior cingulate cortex	cluster 1	HC	51	disease specificity
ROSMAP	posterior cingulate cortex	cluster 2	HC	193	disease specificity
Mayo	temporal cortex		AD	82	external validation, brain region specificity
Mayo	cerebellum		AD	82	brain region specificity

Table **B** shows the results of our analysis. Each row results from training a separate iVAMBN model on each dataset (subset). The Bayes factors for each model (i.e. $\exp(\log(data|modelA)$ - $\log(data|modelB)))$ are reported compared to 1000 randomly permuted network structures on a log_{10} scale. A log_{10} Bayes factor between 0.5 and 1 can be regarded as substantial, between 1 and 2 as strong, and above as decisive [2]. In addition, we also report the overlap of each of the graph structures of each model with that of our primary iVAMBN model. For consistency reasons the overlap was calculated with the previously chosen 40% threshold on the bootstrap confidence, once taking the direction of the edge into account, but also with using the skeleton only, so ignoring the direction of the edges.

Lastly, we analyzed the ability of the primary model to predict the activity score of the prostaglandin module. This was assessed by estimating the Pearson correlation between the respective module's original and predicted activity scores. We report prediction performances within brain regions separately for each cluster of ROSMAP samples due to the observable group effect.

Table B. Specificity to brain region. Column 2: Bayes factor (95% confidence interval) compared to 1000 randomly permuted network structures (log_{10} scale). Column 3: number of edges with bootstrap confidence above 40%. Columns 4, 5: Overlap of graph structure with primary iVAMBN model, including significance ($^* : p < 1e-11$, $^{**} : p < 1e-35$). Here, edges with bootstrap confidence > 40% were considered. Column 6: Correlation (95% confidence interval) between original and predicted prostaglandin pathway based on primary iVAMBN model.

brain region	log_{10} Bayes factor random network	number of edges	overlap with direction	overlap skeleton	correlation prediction
dorsolateral prefrontal cortex (ROSMAP, primary model)	[1.50, 1.54]	163	–	–	[0.79, 0.87]
dorsolateral prefrontal cortex					
(ROSMAP, cluster 1)	[1.30, 1.35]	155	65 (39.88%)*	87 (53.37%)**	[0.55, 0.73]
(ROSMAP, cluster 2)	[1.21, 1.24]	167	62 (38.04%)*	89 (54.60%)**	[0.34, 0.54]
head of caudate nucleus					
(ROSMAP, cluster 1)	[1.42, 1.50]	147	51 (31.23%)*	73 (44.79%)**	[0.25, 0.58]
(ROSMAP, cluster 2)	[1.02, 1.06]	155	58 (35.58%)*	79 (48.47%)**	[0.00, 0.31]
posterior cingulate cortex					
(ROSMAP, cluster 1)	[1.28, 1.41]	138	53 (32.52%)*	71 (43.56%)**	[0.30, 0.66]
(ROSMAP, cluster 2)	[1.13, 1.17]	152	53 (32.52%)*	77 (47.24%)**	[0.27, 0.53]
temporal cortex (Mayo, external validation)	[1.08, 1.15]	137	59 (36.20%)*	78 (47.85%)**	[0.56, 0.79]
cerebellum (Mayo)	[1.09, 1.16]	124	50 (30.67%)*	76 (46.63%)**	[0.38, 0.68]

Disease specificity

Second, similar analyses were done for models trained on healthy control samples from the posterior cingulate cortex, the dorsolateral prefrontal cortex, and the head of caudate nucleus from the ROSMAP study (Table **C**).

Table C. Disease specificity. Column 2: Bayes factor (95% confidence interval) compared to 1000 randomly permuted network structures (log_{10} scale). Column 3: number of edges with bootstrap confidence above 40%. Columns 4, 5: Overlap of graph structure with primary iVAMBN model, including significance ($^* : p < 1e-11$, $^{**} : p < 1e-35$). Here, edges with bootstrap confidence > 40% were considered. Column 6: Correlation (95% confidence interval) between original and predicted prostaglandin pathway based on primary iVAMBN model.

brain region	log_{10} Bayes factor random network	number of edges	overlap with direction	overlap skeleton	correlation prediction
dorsolateral prefrontal cortex					
(ROSMAP, cluster 1)	[1.07, 1.12]	129	51 (31.29%)*	75 (46.01%)**	[0.54, 0.77]
(ROSMAP, cluster 2)	[1.20, 1.23]	171	56 (34.36%)*	90 (55.21%)*	[0.42, 0.60]
head of caudate nucleus					
(ROSMAP, cluster 1)	[1.18, 1.24]	133	55 (33.74%)*	67 (41.10%)*	[0.38, 0.69]
(ROSMAP, cluster 2)	[1.05, 1.08]	154	54 (33.13%)*	83 (50.92%)**	[0.16, 0.44]
posterior cingulate cortex					
(ROSMAP, cluster 1)	[0.67, 0.74]	107	44 (26.99%)*	60 (36.81%)**	[0.47, 0.79]
(ROSMAP, cluster 2)	[1.18, 1.21]	162	53 (32.52%)*	83 (50.92%)**	[0.37, 0.59]

Sensitivity to knowledge graph

To assess the sensitivity of our iVAMBN model against the employed knowledge graph, we performed a random permutation of all edges. We then re-clustered the permuted knowledge graph and re-trained the complete iVAMBN model. After that, we compared the log-likelihood of each patient sample according to the model trained with the randomly permuted knowledge graph with those from the original model (Fig **B**), resulting in significantly lower log-likelihoods for the permuted knowledge graph ($p = 4.14e - 24$).

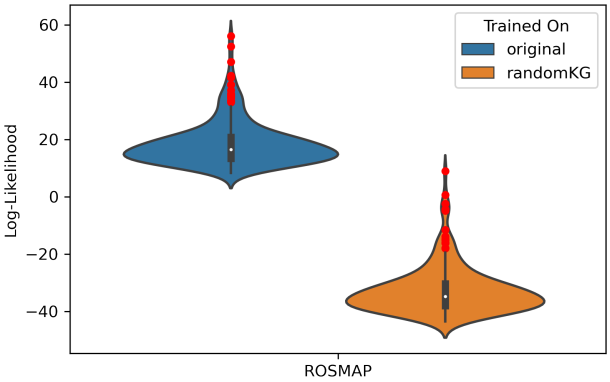

Fig B. Log-likelihoods of each patient sample according to the primary iVAMBN model compared to those of an iVAMBN model trained with a randomly permuted knowledge graph.

References

1. Johnson WE, Li C, and Rabinovic A. Adjusting batch effects in microarray expression data using empirical Bayes methods. Biostatistics 1 2007;8.

2. Kass RE and Raftery AE. Bayes Factors. Journal of the American Statistical Association 430 1995;90:773.
