# Supplementary material for: AI reveals insights into link between CD33 and cognitive impairment in Alzheimer’s Disease
Source: PLoS Comput Biol. 2023 Feb 13;19(2):e1009894. doi: 10.1371/journal.pcbi.1009894 (PMC9956604; doi:10.1371/journal.pcbi.1009894)
Supplement: S4 Note — (PDF) [file pcbi.1009894.s008.pdf]

Supplementary Note S4: Evaluating the Model Fit

To evaluate the fit of the overall iVAMBN model we employed the generative nature of our model: Following a topological sorting of the nodes of the DAG of the MBN we first sampled from the distribution of each node conditional on its parent. Notably, for MBN nodes representing modules this amounted to sample from the posterior of the according HI-VAE. Subsequently, the random sample was then decoded via the HI-VAE. We then compared the marginal distribution of each variable based on the synthetic and the real data. Some distributions are shown in Fig **A**, all other plots are accessible through the github repository.

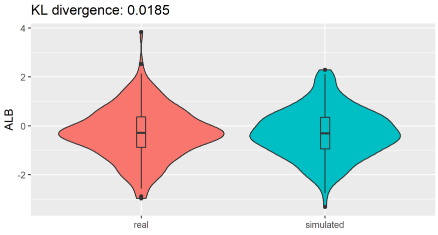

Type	Mean	SD	25%	Median	75%
real	-0.29	1.05	-0.89	-0.29	0.36
simulated	-0.3	1	-0.95	-0.31	0.35

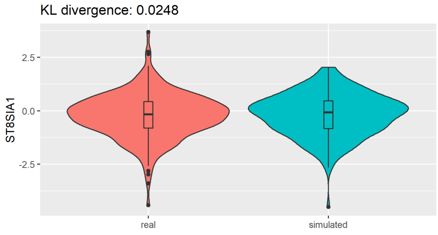

Type	Mean	SD	25%	Median	75%
real	-0.22	1.06	-0.81	-0.17	0.43
simulated	-0.19	1	-0.83	-0.07	0.46

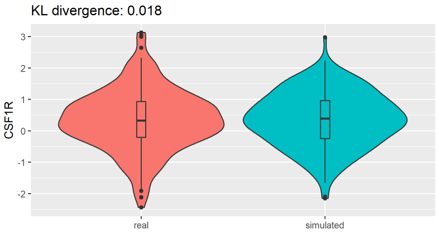

Type	Mean	SD	25%	Median	75%
real	0.34	0.97	-0.2	0.33	0.93
simulated	0.36	0.88	-0.25	0.39	0.97

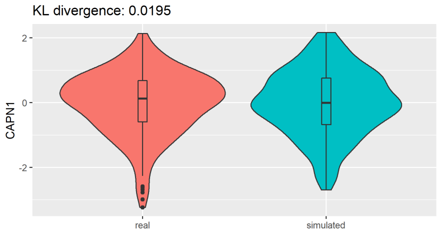

Type	Mean	SD	25%	Median	75%
real	-0.02	0.98	-0.59	0.13	0.68
simulated	-0.01	1.01	-0.68	-0.01	0.76

Fig A. Distribution of single features in real data versus simulated data.

Furthermore, we compared the correlation matrices of synthetic and real data (cf Fig **B**). The relative error between the real and simulated values was 0.7138.

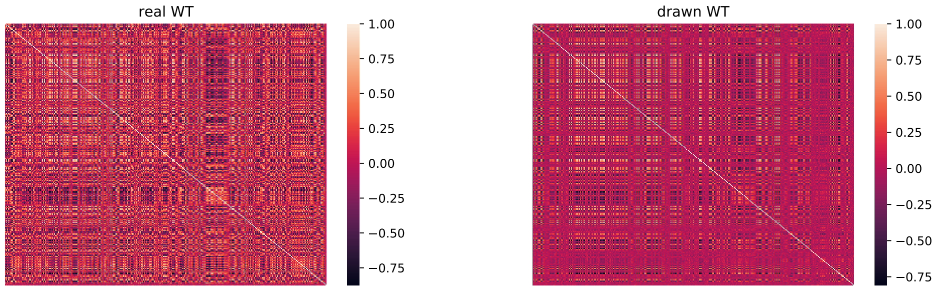

Fig B. Correlation matrix of real and drawn data.
