# Supplementary figures and images for: AI reveals insights into link between CD33 and cognitive impairment in Alzheimer’s Disease
Source: PLoS Comput Biol. 2023 Feb 13;19(2):e1009894. doi: 10.1371/journal.pcbi.1009894 (PMC9956604; doi:10.1371/journal.pcbi.1009894)

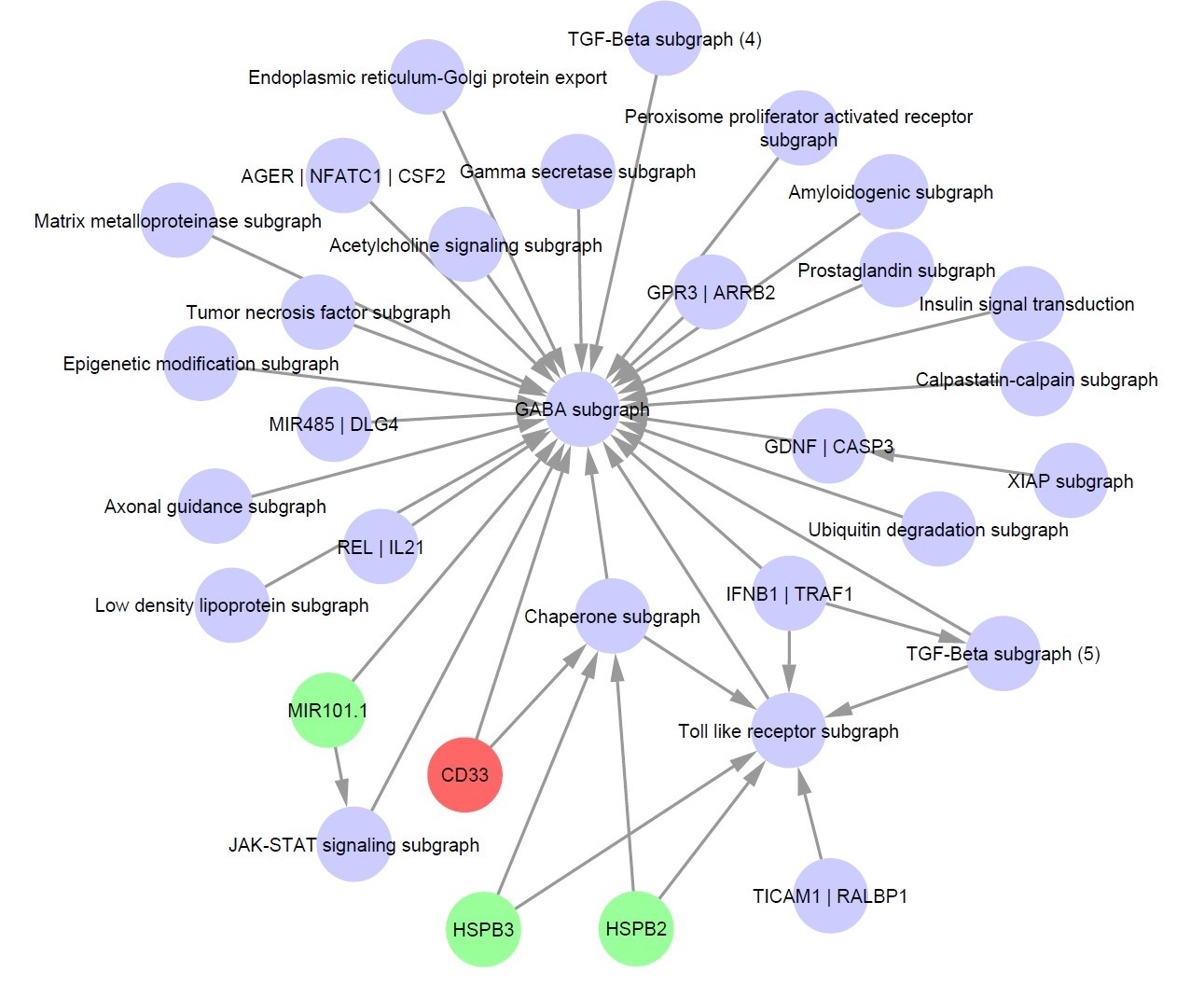

Supplement: S1 Fig — Knowledge graph modules (clusters) are annotated with significantly enriched (adjusted p < 0.05) NeuroMMSig mechanisms. If the genes in a module do not enrich NeuroMMSig terms significantly, symbols of contained genes are reported. If multiple significant enriched terms could be found, the most significant pathway was used for naming the corresponding node. In case that a module contains a single gene, the gene symbol is reported. CD33 is marked in red, while other single genes are displayed in green, and non-single gene modules in purple. (PNG) [file pcbi.1009894.s009.png]

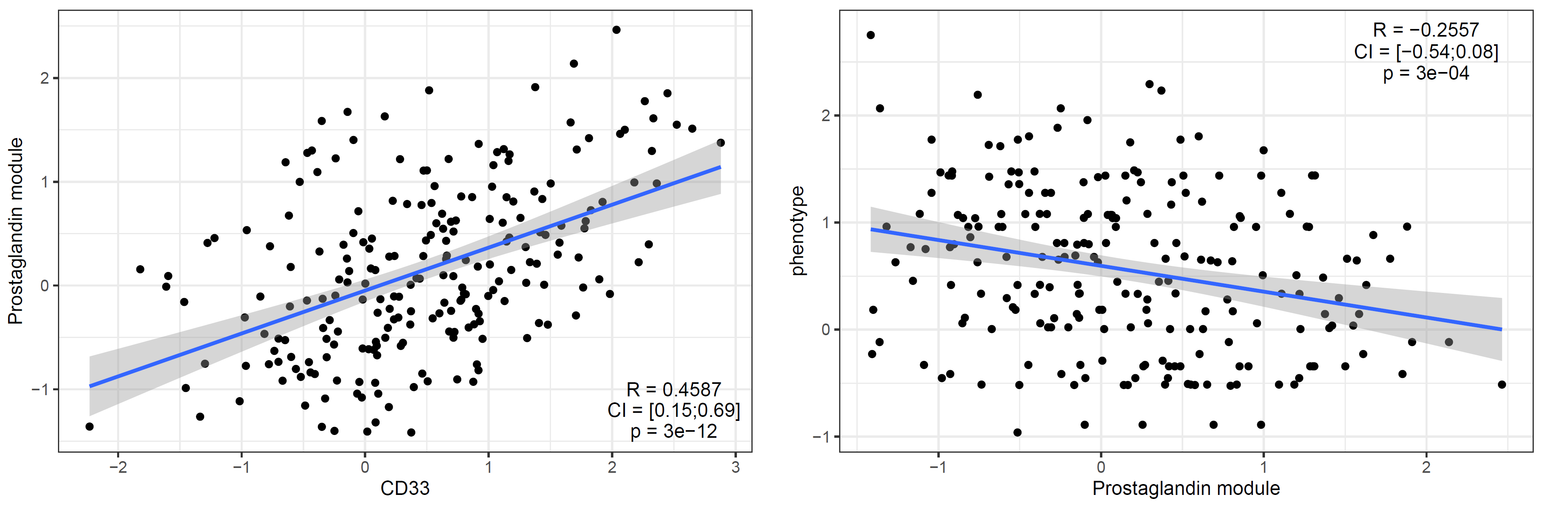

Supplement: S2 Fig — Each correlation (R) is shown along with its confidence interval (CI) and multiple testing adjusted p-value. Left: Correlation of CD33 with prostaglandin pathway module. Right: Correlation of prostaglandin pathway module with the phenotype module. (PNG) [file pcbi.1009894.s010.png]

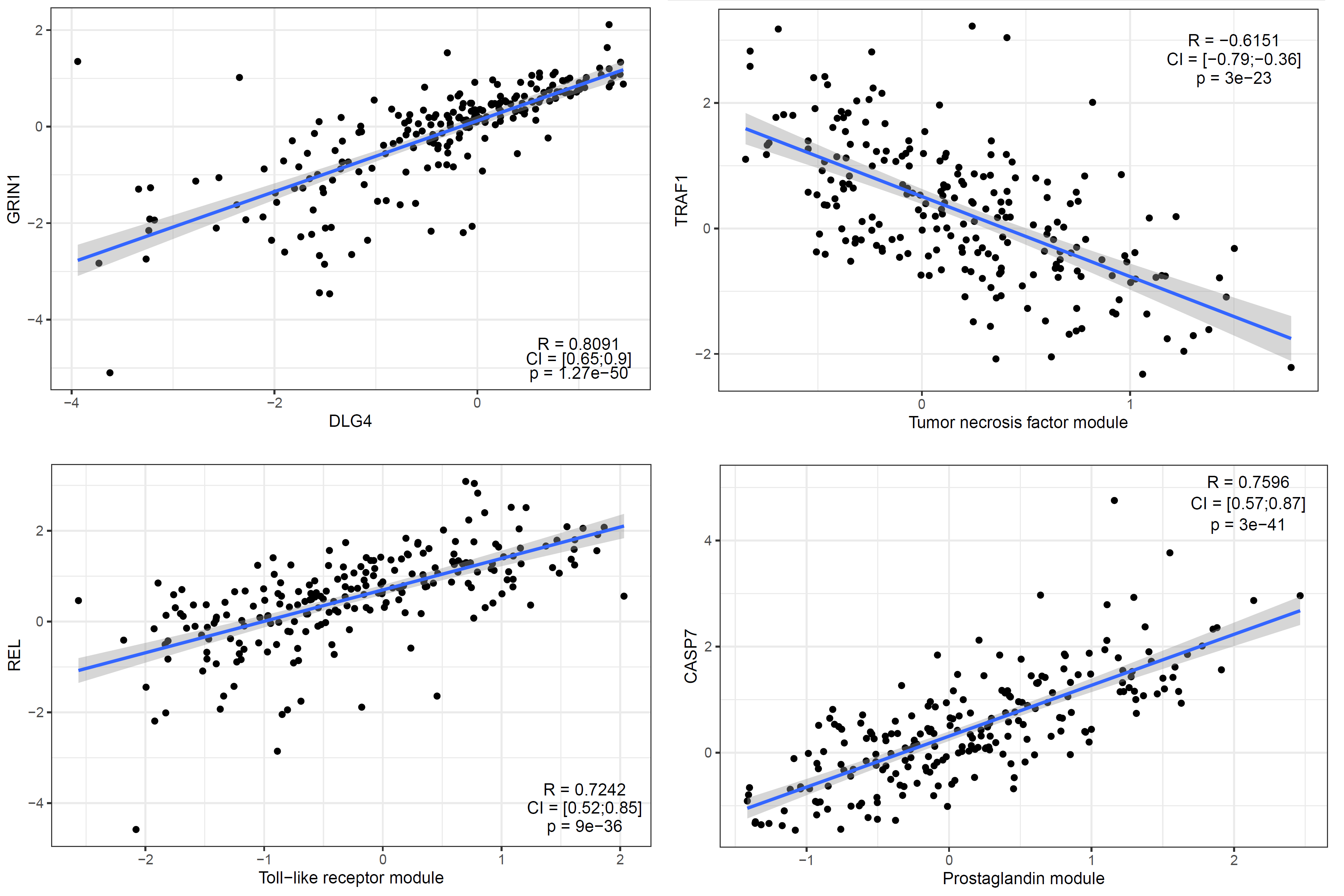

Supplement: S3 Fig — Each correlation (R) is shown along with its confidence interval (CI) and multiple testing adjusted p-value. The from module is always shown on x-axis while the to module is shown on y-axis. (PNG) [file pcbi.1009894.s011.png]

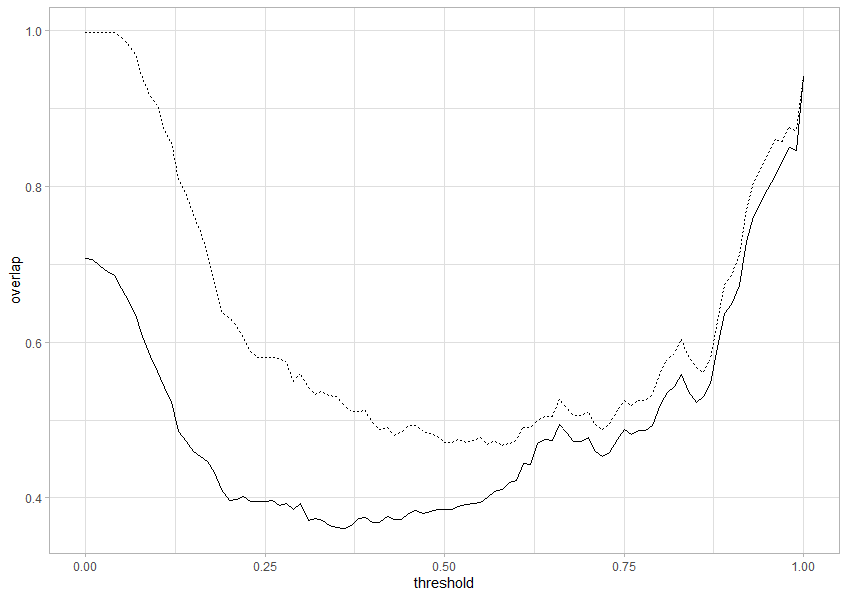

Supplement: S4 Fig — The overlap of the independent bootstrap structure learning for ROSMAP data and Mayo data is shown for different threshold values. The black line represents the overlap when considering the direction of the edge, the dashed line the overlap of the network skeletons. (PNG) [file pcbi.1009894.s012.png]

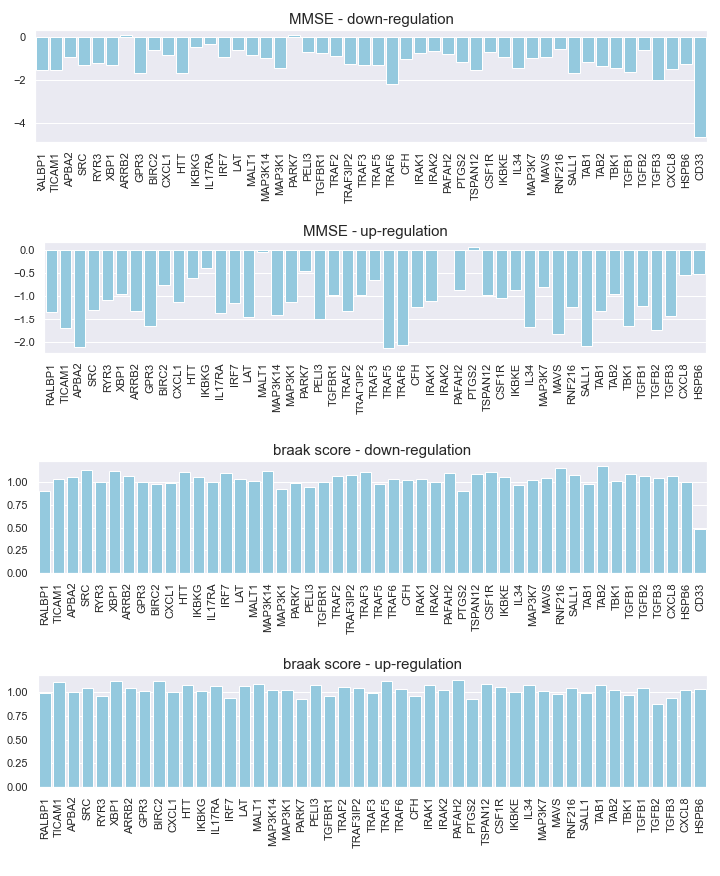

Supplement: S5 Fig — The bar plots show the difference between the mean score in the original data and the mean score in the simulated data for each target and each phenotype score, namely MMSE (upper two rows) and Braak score (bottom two rows). First and third row shows the results of under-expression, while second and forth rows shows the results of over-expression. (PNG) [file pcbi.1009894.s013.png]
