# Supplementary material for: AI reveals insights into link between CD33 and cognitive impairment in Alzheimer’s Disease
Source: PLoS Comput Biol. 2023 Feb 13;19(2):e1009894. doi: 10.1371/journal.pcbi.1009894 (PMC9956604; doi:10.1371/journal.pcbi.1009894)
Supplement: Attachment — Submitted filename: Response-to-reviewers-iVAMBN.docx [file pcbi.1009894.s014.docx]

**Dear Lars Juhl Jensen,**

**We would like to thank the editor and reviewers for their careful and useful evaluation of our manuscript. We believe that we addressed all comments. Please find our point-by-point response to the reviewer comments below. In response to the comments/questions, we have marked our responses with an asterisk mark (“*”) and highlighted changes within the manuscript to make our responses easy to find.**

**We very much hope that we addressed all issues raised about our manuscript and that it is now acceptable for publication. Thank you for your time.**

Reviewer #1:

I'm enthusiastic about your goal of doing in silico screening of the effects of perturbing specific genes, and I think you've assembled a nice array of technologies for doing that. However, there are limitations of the implementation, and more to the point, after reading this several times I really couldn't tell what you were doing on several points - the take away from this is that if someone who cares way more about figuring out your methods than a casual read does, there are indeed issues. I've tried to make specific questions and points suggestions about this.

**** We tried to clarify our approach by re-designing Figure 1 and re-writing several parts of the Results and Methods Sections. We also would like to point out that we have made the code of our analysis available on a gitlab repository.**

Need refs for sentences 1&2.

**** Thank you, the references were added to the manuscript.**

I'm sure you know what you did, but the use of "module" and "cluster" in multiple contexts or with distinct contents can be (is) confusing when you don't clearly note what type of cluster of module you're referring to. Deploying a simple descriptor, as in "we used these [gene] modules in tandem with the clusters [of proteins] in order to....." where you would insert the modifier in []. This situation comes up so many times, so please examine every usage of those words and clarify.

**** We have tried to make our terminology more consistent. Additionally, we rephrased some sentences to make it clearer what each module consists of.**

Right around line 108 there should be a statement of why you're deploying VAMBN, other than just you have it on hand.

**** There were several considerations why we chose VAMBN: We wanted to develop a quantitative network model integrating qualitative background knowledge presented by a graph with patient-level clinical and gene expression data. Due to the lack of detailed knowledge about biochemical reactions and the lack of time series data, the development of an ODE based mathematical model was out of question. Hence, we focused on the area of probabilistic graphical models and more specifically Bayesian Networks. However, standard Bayesian Network implementations require normally distributed or multinomially distributed data, which is not the case in our situation, at least not for the phenotype part of our data. Furthermore, it should be seen that structure learning of Bayesian Networks is an NP hard problem, where the number of possible network structures grows super-exponentially with the number of nodes in the network. Hence, modelling each gene as an individual node in a Bayesian Network would raise severe concerns regarding structure identifiability.**

**To address all these points, we employed a modular Bayesian Network approach, which is suitable for modelling multi-modal and heterogeneous data. VAMBN is to our knowledge the only method that can do that.**

**We have added a corresponding explanation to the Introduction Section in our manuscript now.**

How specific are these results to cd33? Do you get similar ones if you put other genes in the microglia module through this?

**** Our knowledge graph was built around CD33, because our project focused on a better understanding of CD33 as a potential drug target. We do not claim that our model reflects the entirety of AD biology. While our method is in principle applicable to other potential targets (including other microglia targets), we lack the data to validate according simulation results. We only have experimental data of a CD33 perturbation. But we have now added additional simulation results on other possible targets. Of course, it is impossible to validate those results due to the lack of experimental data.**

Is the approach fast enough that you can use it for screening? i.e. put every gene through it to prioritize disease molecular systems

**** Once an iVAMBN model has been learned, the simulation of counterfactual scenarios, such as the down-expression of a specific gene or an entire gene module, is fast enough to be used for screening. But one would have to invest time for implementation of an automated analysis pipeline of simulation results.**

What is the false positive rate associated with table 3?

**** The last column in Table 3 indicates, whether simulations and experimental findings both agree with respect to significance / non-significance (+), or whether there was a contradiction (-). As you can see, there were 3 / 22 false positive predictions by our simulations, if you accept a false discovery rate threshold of 5%. Notably one of the false positives (TICAM1 / RALBP1) had an adjusted p-value of 5.6% in the experimental data.**

**We have added a corresponding remark to our paper now.**

Considering the significance calculation of the similarity of the bayesian networks from the two cohorts - wonder if that's realistic as they have the same underlying the same knowledge graph, so is it an independent replication?

****We performed two types of validation: First, we checked whether the model trained on ROSMAP could explain the independent data from the Mayo study. Second, we checked the overlap of graph structures of iVAMBN models learned independently on ROSMAP and Mayo datasets. We have tried to make this point clearer now.**

There are single genes listed in table 3 - is that really a "mechanism"?

**** We have changed our wording now and avoid the word “mechanism”.**

You're being pretty restrictive with AD if you only end up with 221 samples in rosmap - I'd want to know exactly what your criteria was for that - there are ~1600 dlpfc samples available and we show that most brains have some level of AD pathology. Do you know conclusively that the AD graph is indeed somehow specific to AD brains? Why not run it on some definition of healthy and see how it differs? You could also get data from at least three other regions in ROSMAP with several hundred gene expression arrays - would be a big boost to validating your model.

**** We use the data from the RNAseq Harmonization Study. We made this clearer now by referencing the respective Synapse location in the Data Availability statement. We equally adapted the Acknowledgement section for that. The given count file for ROSMAP from the RNAseq Harmonization Study contains 640 samples of which 632 contained the covariates required for our analysis. We only used samples with an AD diagnosis, resulting in 221 samples. As mentioned in Section “Gene Expression Analysis” samples from cognitively normal patients were used to convert gene expression data of these AD samples into abnormality scores for each gene. This was done to ensure gene expression data to lie on a comparable numerical range in ROSMAP and Mayo. However, for the same reason it is not possible to run our analysis with only using healthy controls, because then we would miss a reference for standardization.**

**We focused our modelling only on AD samples of specific brain regions in ROSMAP to have the possibility to validate our findings with Mayo.**

How does the data reduction in the inner layers of the autoencoder compare to the conditional dependencies of bayesian networks?

**** The autoencoders learn a low dimensional (conditional) Gaussian distribution of a group of variables (= modules), whereas the Bayesian Network models the statistical dependencies between modules. Hence, autoencoders and Bayesian Network complement each other.**

Why don't you incorporate the score of the networks in addition to edge frequency?

**** The marginal log-likelihood of an iVAMBN model log p(data | model) is any real number, which cannot be interpreted easily. This number tells you only something about the ability of the overall model to explain the data. But it does not tell you something about the robustness of the model, i.e. how frequent individual edges are re-identifiable when subsampling the data. Hence, it is necessary to also check, how frequent edges are re-identified from data when performing a bootstrap.**

**Both, edge frequency and overall network score (i.e. log p(data | model) express something different and cannot be combined.**

What is your edge updating function for the bayes net?

**** Structure learning for the Bayesian network is done with hill climbing algorithm using the Bayesian Information Criterion (BIC) as a scoring function, as stated in Section “Model Training” and Supplementary Note S2.**

Not clear if you're training an encoder per each cluster of genes in the knowledge graph or treating each cluster as a single variable, and then the autoencoder handles this group of meta variables.

**** We train an encoder per defined module. This is already clearly stated in the manuscript under Methods > Quantitative Modeling Across Biological Scales via iVAMBN > Model Training and now hopefully also clearer from the workflow figure.**

Should provide some guidance on the primacy of gene expr vs knowledge graph and how that stems from structure of algo

**** As explained in Section “Model Training”, the knowledge graph is effectively only used to initialize the structure learning algorithm and to pre-define / white list certain edges. This choice was made based on a data driven comparison of different knowledge integration strategies via cross-validation, see Sections “Integrative Variational Autoencoder Modular Bayesian Network Model” and “Model Training”. That means iVAMBN is principally free to add edges to the knowledge graph, if the data provides according evidence.**

When you say AD-specific mechanisms L126, do you really mean it? i.e. if a gene was ever implicated in some other disease you'd exclude it?

**** “AD-specific” referred to the disease focus of the gene-set collection (NeuroMMSig). Other gene-set collections, e.g. KEGG, are mostly not designed for one disease but for a general purpose. It is not meant that genes implicated in other diseases have been excluded. We tried to clarify this aspect now by replacing “AD specific” with “AD associated”.**

Need to set up the integration of knowledge graphs and expression with some context - ideally data driven. i.e. what is the average coexpression value for various knowledge graphs? Are we combining two data sources with some similarity or no similarity greater than chance?

**** We do not fully understand this question. As mentioned above, we used the clustered knowledge graph to initialize a Bayesian Network structure learning algorithm and to pre-define / white list certain edges. So, our approach is indeed data driven, but informed by prior knowledge. Once again, please note that we compared different knowledge integration strategies in a data driven manner using cross-validation.**

Why did you chose markov clustering for the K-graph? That's basically the method that always loses in most clustering methods comparisons:) I'd want to see at least one more clustering method applied and equivalent results coming out, as well as some notes on the modularity of the partition you find.

**** This is a good point. We now performed a broader comparison of different graph clustering algorithms.**

Not defined in fig 1 if knowledge graph has green and blue balls denoting genes or if those are modules of genes. What are BL and WL?

**** We have completely re-designed the workflow figure and hope that these points are clearer now.**

l133 "here: gene clusters and phenotype related scores," do you mean that there are genes AND phenos in the same module or they are each in their own module?

**** No, we mean that there are modules comprising genes, and there is a module containing phenotype related scores. We have re-formulated this aspect in the manuscript and hope that it is clearer now.**

Figure 1 caption "constraining potential connections between them" do you mean only connections between groups of variables or between pairs of variables within a group or both?

**** The knowledge graph is only used to inform the structure learning algorithm about potential connections in the Bayesian Network. Nodes in the Bayesian Network correspond to modules, i.e. groups of features. Hence, the prior knowledge (we omitted the word “constraint” now) only relates to connections between those modules. We made this clearer both in the figure caption and the manuscript.**

Figure 1 design needs to be revamped. You know what you did, and I'm sure the fig is one accurate depiction of that, but if you don't know what you did, it's not sufficient to figure out what you did.

**** Thank you for this feedback. We have re-designed Figure 1 describing the workflow and hope that it is clearer now.**

Reviewer #2: Summary:
An interesting approach to understanding mechanisms of Alzheimer's disease that blends AI-based data-driven feature generation with knowledge graphs. Experiments included both human and mouse data.

Details:
Apologies for the brevity of this review. Time is precious.

Conceptually the method is sound and the implementation as described is appropriate and understandable. Briefly, input features (genetics, biomarkers, clinical) are mapped into modules using clustering plus some manual editing. A VAE is used for dimensionality reduction of the modules into a Gaussian or multinomial parametrization, which become the nodes of a BayesNet, with network parameter learning constrained by a knowlege graph and a few sensible causality constraints.

Cross-cohort harmonization was used to adjust for batch effects in the human data.

The method is an intelligent augmentation of the authors' existing VAMBN to incorporate existing knowledge into data-driven modelling.

The authors make grand/general statements about their model (don't we all...) and the possible ramifications for identifying drug targets, but demonstrate only one result on the possible effects of CD33 downregulation. I suspect that this was likely due to the availabiltiy of mouse data for validation, but I think the reader would benefit from an additional results section where the authors analyse other potential targets, and discuss them in the context of the literature. In particular, it would be valuable to consider two categories of targets: 1) well-studied therapeutic targets such as those related to existing GWAS studies and/or clinical trials (e.g., of anti-amyloid and anti-tau drugs); 2) potential novel targets. This would add considerable value to this study, and generate much discussion in the field.

**** Thank you for these suggestions. We have now added additional simulation results to the supplements of our paper on all possible targets in the model that make sense to simulate. Genes belonging to modules which are not an ancestor of the phenotype module are excluded, as those will not have any effect on the phenotype by definition. Of course, it is impossible to validate those results due to the lack of experimental data.**

I appreciated the method's aim to circumvent the need for fitting a differential equation model. But I wonder if there is any scope for using longitudinal data to validate the model — if such data exists, of course.

**** In addition to the phenotypic characterization, we use gene expression data from post-mortem gene expression data. These data are only static, of course. So, longitudinal modelling is not possible based on the given data.**

= Comments =
Methods: It makes sense to retrain the ROSMAP model for validation on the Mayo dataset. What is not clear from the manuscript is the consistency of the two trained models (with and without MMSE) and analysis of any differences. Suggest that the authors provide results of an analysis that compares the two ROSMAP-trained models, e.g., edge strength differences (if any).

**** This is a good point. We added a comparison of the network structures learned from both datasets now.**

Results: CD33 down-expression simulation results are interesting. The discussion around caveats (starting on line 311) raises questions. Primarily, the potential safety issues - have the authors considered including such issues as additional constraints in the model? For example, explicitly excluding unsafe targets for therapy from the knowledge graph seems sensible (but may have implications I haven't thought of). I (and the readers of this manuscript) would be very interested in the authors' thoughts on the matter.

**** Thank you for this interesting question. We believe that it could be possible to add known side effects of approved drugs targeting a specific protein to our model’s graph structure. This could provide hints on possible side effects. We have added a corresponding remark as an outlook to future research to our discussion.**

Further comments and suggestions are below.

- Line 160: A github repository can be deleted, so the full list of molecules within each cluster should be included in Supplementary Material.

**** We have added the list of molecules to the Supplementary Material.**

- Line 193: Please explain the rationale behind your choice to use 40% as the threshold.

**** We agree that any threshold appears a bit arbitrary. But our aim was to focus our visualization and discussion on edges that are found rather statistically stable. At the same time, we also wanted to visualize and discuss the CD33 🡪 prostaglandin and prostaglandin 🡪 phenotype edges, which have a frequency just below 50%. Therefore, we chose the 40% threshold.**

**To be more transparent we have now added an Excel sheet with all edges (without applying any threshold) to the Supplements.**

- Line 216, Supplementary Figure S3: A linear association is surely not suitable for MMSE. From the data it is apparent that the MMSE ceiling is undersampled, but the floor appears to be well sampled. What about fitting a sigmoid instead?
**** This is true. Given the fact that the MMSE score is a member of the phenotype module and thus transformed non-linearly via the autoencoder we decided to depict the correlation with the phenotype score instead. We think this provides a more realistic impression.**

- Figure 5: A typo in the caption: I think "results in higher scores" should be "results in lower scores".
- Throughout: remove "highly significant". Statistical significance thresholds are arbitrary.
- Throughout: give it another proof reading, some typos exist.

**** We gone over the manuscript and have hopefully removed all typos now and removed the “highly significant”.**
