# Supplementary material for: AI reveals insights into link between CD33 and cognitive impairment in Alzheimer’s Disease
Source: PLoS Comput Biol. 2023 Feb 13;19(2):e1009894. doi: 10.1371/journal.pcbi.1009894 (PMC9956604; doi:10.1371/journal.pcbi.1009894)
Supplement: Attachment — Submitted filename: Response-to-reviewers-iVAMBN-revision2.docx [file pcbi.1009894.s015.docx]

**Dear Lars Juhl Jensen,**

**We would like to thank the editor and reviewers for their careful and useful evaluation of our manuscript. We believe that we addressed all comments. Please find our point-by-point response to the reviewer comments below. In response to the comments/questions, we have marked our responses with an asterisk mark (“*”) and highlighted changes within the manuscript to make our responses easy to find.**

**We very much hope that we addressed all issues raised about our manuscript and that it is now acceptable for publication. Thank you for your time.**

Reviewer #1:

Dear authors,
I appreciate the authors responses to these critiques, and I think the paper is resultantly clearer and will reach a broader audience.

A few large-scale issues remain:

Network generation:
There's a relatively easy way to increase the accuracy of your Bayesian networks, which would be to use REV-move or some other more advanced edge updating rule, because as you're aware, it's very easy for structure-move to become stuck, particularly in such large networks.  There are implementations of this tested on gene expression data that showed several times the accuracy of structure-move, so this is a relatively easy way to drastically improve accuracy - particularly important, again, in light of the easy with which struct-move gets stuck.

**** Thanks for this recommendation. As our data is multinomial and normally distributed only few structure learning algorithms are applicable. But we have now integrated a broader comparison of the structure learning algorithms that are implemented in the bnlearn package. The results can be found in the Supplementary Material (Note S2). We would like to mention that the issue of local minima in structure learning cannot be entirely circumvented by any existing algorithm.**

Validation:
The Mayo validation is helpful.  However, there are other datasets which are actually larger (n>1000) and from other brain regions that will allow you to much more accurately estimate networks.  Just go to the RADC website and request gene expression data - it's all freely available.  For modern omics studies I would expect to see such tests ,as the data is easily available, and it will go a long way to convincing readers of the reliability of the method.

**** Using a validation data set with data coming from different brain regions wouldn’t be comparable to our current model, because gene expression is known to be brain region dependent. We have checked the RADC website, but it re-directs us to the ROSMAP dataset which we already have used as the training dataset for the iVAMBN model.**

Model components:
One of the most intriguing aspects of this work is the integration of prior knowledge in the form of a graph.  Opinions about the validity of such knowledge sources vary, so it would be helpful to see to what extent this helps to increase predictive accuracy.  Ideally, the knowledge graph would be completely ablated and then predictive accuracy reassessed in all aspects.  If that is not possible, it should be possible to, say, delete 25, 50 and 75% of the edges in the graph and rerun to assess its contributions.  Similarly, a varying number of incorrect edges could be added to the graph.  This would provide an estimate of the sensitivity of the knowledge graph to new information, as well as it's total contribution to predictions.

**** Indeed, the increase in prediction when integrating a knowledge graph in the model construction, is interesting to estimate. We had previously already included such an analysis of different methods of integrating the knowledge graph vs using a completely data driven approach in the Supplementary Material (Note S2). We have extended this analysis now to the different structure learning methods mentioned before.**

Screening:
I think many users of your work will be interested to sift through the membership of a gene list, based on their importance, as determined by your method.  This seems to entail just putting a for loop around your method, grabbing the knowledge graph from ref 10, or a sub graph, and rerunning.  As motivation, I've had multiple pharma specifically ask me not just to reiterate known targets (i.e. cd33), but want info on what a given computational method has predicted that is not already popularly supported.  In summary, I think it would be very useful to share the screening results for all microglia genes - it would demonstrate this is feasible and provide directly useful results.

**** We have simulated the up- and down-regulation of all potential drug targets used in the model. The results are described in the Result section and a plot visualizing those results is included in Supplementary Figure S7. So, anyone can go through it and check their potential target. If a target is not in the list, it was either not considered in the model, or was estimated to have no effect on the phenotype through the Bayesian network.**

Minor:
For the cd33 KO data, if you look at the genes which did not match your prediction, do they share some common feature?  This might enable you to comment on some biological process which is outside of the scope of the method (possibly for understandable reasons).

**** Our analysis on the matching of the model’s prediction with experimental data was done by comparing the predicted change of a module’s activity score to the observed changes of genes assigned to the same module. More specifically, we used a global test to analyse the overall / coordinated differential expression of genes assigned to each particular module. That means we interpreted all genes assigned to a module as a gene set. The details are described in Section “CD33 knock-down expression and analysis” in the main paper, and results can be found in Table 3.**

**Based on these findings it can be, for example, seen that the model’s predictions do not match with the experimental cell line data for the TGF-beta pathway, but they agree e.g. well for genes involved into acetylcholine signalling.**

**Taken together we believe that the results presented in Table 3 indeed allow for understanding biological commonalities of genes, which did not match our predictions.**
