# Supplementary material for: AI reveals insights into link between CD33 and cognitive impairment in Alzheimer’s Disease
Source: PLoS Comput Biol. 2023 Feb 13;19(2):e1009894. doi: 10.1371/journal.pcbi.1009894 (PMC9956604; doi:10.1371/journal.pcbi.1009894)
Supplement: Attachment — Submitted filename: Response-to-reviewers-iVAMBN-revision3.docx [file pcbi.1009894.s016.docx]

**Dear Lars Juhl Jensen,**

**We would like to thank the editor and reviewers again for their careful and useful evaluation of our manuscript. Please find our point-by-point response to the reviewer comments below. In response to the comments/questions, we have marked our responses with an asterisk mark (“*”) and highlighted changes within the manuscript to make our responses easy to find.**

**We very much hope that we addressed all issues raised about our manuscript and that it is now acceptable for publication. Thank you for your time.**

Reviewer #1: Thank you for your responses and additions to the text to date.  Below are the major remaining issues.

For the knowledge graph, what if you use something else (louvain or other mainstream options) other than markov clustering (which does not fare well in comparisons of clustering methods) - how much do the modules change and how much do those changes impact performance.  Would anticipate that the exact choice of algorithm shouldn't affect results, but if it does, would be important to note this sensitivity.

**** As explained in the Methods part of our paper we systematically compared three different popular graph clustering algorithms: Markov clustering, infomap and edge betweenness (Girvin-Newman clustering), and results are detailed in Supplementary Table 7. Based on the results we decided for Markov clustering. We cite the corresponding text passage:**

***“For identifying modules of genes we clustered the knowledge graph with the help of different graph clustering algorithms:***

- ***Markov Cluster algorithm implemented in the MCL- package in R.***
- ***edge betweenness community detection implemented in the R package igraph***
- ***infomap community finding method implemented in the R package igraph***

***After clustering, genes being part of a single cluster were assigned to a corresponding module. Genes being not clustered but only connected to one cluster, were merged into that cluster. Genes being connected to multiple clusters were kept as single gene modules (modules consisting of a single feature) for further analysis.***

***We selected the best clustering algorithm according to multiple metrics described in [58]*** ***including internal density, number of edges inside clusters, average degree, expansion, cut ratio, conductance, and norm cut. Based on these metrics the average ranking of each graph clustering algorithm was computed with the rational in mind, that each cluster should have a high internal density and sparse connections across clusters. This resulted in choosing the Markov clustering algorithm for further analyses. The metrics for each clustering algorithm can be found in Supplementary Table S6.”***

**As indicated by the results presented and Supplementary Note S2 our analysis is indeed affected by the infusion of the module graph (= clustered knowledge graph).**

**We added an analysis based on a randomly permuted module graph. The results of this analysis underline the influence of the module graph on the model, as we could observe a significantly lower marginal log-likelihood when using the randomly permuted knowledge graph, which is shown in Supplementary Figure S8 (see also below).**

As the model has several major components, helpful to know to what extent each contributes to the prediction.  For instance, if you scramble the edges in the knowledge graph, cluster that and use it, how much does accuracy fall?

**** We performed this experiment and it shows – as expected – a dramatic decrease in the marginal log-likelihood, i.e. the experimental data cannot be explained. This is now described in the Supplementary Note S3 and shown in Supplementary Figure S8 (as shown above).**

The titular claim is that this is a network for Alzheimer's disease.  You restrict your subjects down to 200, but by using all the DLPFC data you would have 1200+samples, and it's not been proven that the resulting network from that would be different from this one.  Indeed, many cognitively healthy subjects will have brain pathology.  You can either try to prove that the network is AD-specific, or just include a broader range of samples to increase the accuracy of the findings or perform cross-validation.  In either case, you will need to explore the full expression data set.

**** We would like to mention that we have not just run cross-validation in the primary analysis, but we performed a full external validation of our iVAMBN model with data from the Mayo study.**

**Based on the requirements of the reviewer we have now trained additional iVAMBN models on available data from other brain regions (AD patients) as well as data from healthy controls (HC subjects) from both, ROSMAP and Mayo studies. We have added a corresponding Section “Specificity and Sensitivity of iVAMBN model” to our manuscript now. Full results are included in Supplementary Note S3 (“Sensitivity and Specificity Analysis”).**

**Briefly, our analysis indicates that there are significant overlaps on the graph structure with iVAMBN models trained on different brain regions, but the degree of overlap differs. Moreover, the degree of overlap is clearly lower, if we use data from HC subjects for training.**

**Altogether we do not claim that our model is entirely specific for AD, and we have also replaced “AD specific” by “AD focused” throughout our manuscript.**

I am puzzled by the statement that "brain expression is known to be region specific" as a reason not to repeat this analysis with data from other regions - if anything, that is a reason TO test it in other regions. In any case, it's very standard to repeat tests in all regions - even if you think it will not be replicated - in order to see how the results vary across regions. You can see that several hundred samples are available in ROSMAP for PCC and AC, as well as ~200 in the hypothalamus and nucleus accumbens. Furthermore there are 1200+dlpfc samples available as mentioned previously. Testing in all of these data are essential to measuring the robustness of the findings. I've asked that these be made more obvious on the Rush resource page, but in any case just enter a limited data request DUA and in text say you're requesting gene expression data for all of these regions and you'll receive it.

**** We have checked the available data again. First, we would like to mention, that we employed data from the RNASeq Harmonization study (**[**https://www.synapse.org/#!Synapse:syn21241740**](https://www.synapse.org/#!Synapse:syn21241740)**).**

**The reason is that the data from this study is aligned in terms of the processing of the samples from ROSMAP and Mayo, which will exclude unwanted effects in the analysis due to differences in the sequencing approach and pre-processing of the samples. Secondly, we need to state that at the beginning of this work only the first batch of ROSMAP was available in the RNAseq Reprocessing study (**[**https://www.synapse.org/#!Synapse:syn9702085**](https://www.synapse.org/#!Synapse:syn9702085)**). We indeed missed to check whether there was any update of the data before preparing the final manuscript. We have now used other available samples for an additional specificity analysis for our model, as explained in the answer to your last question. The results are now shortly described in the main text and in the Supplementary Note S3 in more detail.**

**As indicated in our answer to the last question, we applied the same strategy also to data from cognitively normal patients. A more extensive description of which samples were used can now be found in Supplementary Note S3 and Table S4.**

**We hope that with that explanation and additional analyses all your questions are answered and that the additions made to the manuscript are helpful for understanding what we did and how we validated the model.**
